# Protocol for a randomized pilot study (FIRST STEPS): implementation of the Incredible Years-ASLD® program in Spanish children with autism and preterm children with communication and/or socialization difficulties

**DOI:** 10.1186/s13063-021-05229-1

**Published:** 2021-04-20

**Authors:** Fátima Valencia, Elena Urbiola, Marina Romero-González, Inmaculada Navas, María Elías, Alexandra Garriz, Almudena Ramírez, Laia Villalta

**Affiliations:** 1 Department of Child and Adolescent Psychiatry, Institute of Psychiatry and Mental Health, Hospital General Universitario Gregorio Marañón, Madrid, Spain; 2 Maternity Hospital, Hospital Regional Universitario de Málaga, Málaga, Spain; 3 Department of Brain Health, CIMES, Faculty of Medicine-IBIMA, UMA, Málaga, Spain; 4 Department of Psychiatry and Psychology, Hospital Sant Joan de Déu de Barcelona, L’Hospitalet de Llobregat, Spain; 5 Children and Adolescent Mental Health Research Group, Research Institute Sant Joan de Déu, Esplugues de Llobregat, Spain

**Keywords:** Parenting intervention, Pilot, ASD, Preterm children, Child development, randomized controlled trial, Complex intervention

## Abstract

**Abstract:**

Having access to parenting interventions in the early years is key to improve the developmental outcomes of children with neurodevelopmental problems. The Incredible Years® (IY) Parent Program is a group intervention that has demonstrated efficacy in terms of reducing stress in parents, as well as improving behavioral, emotional, and social outcomes in children. The program has been recently adapted for families of children with autism or language delays (IY-ASLD®). This intervention has not yet been implemented in the Spanish Public Health System, where there is a scarcity of evidence-based interventions being offered to families with young children presenting neurodevelopmental problems. The main aims of this study are to determine the feasibility of implementing the IY-ASLD® program within Spanish Child Mental Health Services and to examine parents’ acceptability and satisfaction with the intervention. As a secondary objective, we aim to evaluate its preliminary effectiveness in terms of reducing parental stress and behavioral difficulties in their children. The FIRST STEPS study is a multicenter, pilot randomized controlled trial comparing the IY-ASLD® program with a treatment-as-usual (TAU) condition. Approximately 70 families of children with autism spectrum disorder (ASD) and preterm children with communication and/or socialization difficulties (aged 2–5 years) will be recruited. Families will be assessed prior to randomization and after the intervention. Due to the COVID-19 pandemic, the intervention will consist of 22 weekly online sessions (approximately 6 months).

The FIRST STEPS pilot trial will demonstrate the feasibility and acceptability of reliably implementing the IY-ASLD® program within the Spanish Public Health System. The results of this study could represent the first step to inform policymakers in Spain when designing evidence-based healthcare pathways for families of children presenting ASD symptoms or neurodevelopmental difficulties at early stages.

**Trial registration:**

ClinicalTrials.gov NCT04358484. Registered on 04 April 2020

## Introduction

Autism spectrum disorder (ASD) is a neurodevelopmental disorder involving social communication disturbances and a restricted pattern of interests, present before age 3 [1].

Preterm children are at particular risk of presenting a broad range of developmental difficulties, such as language delay, communication and social disturbances, and ASD [2–4]. Both children with ASD and preterm children are more likely to exhibit internalizing and externalizing difficulties early in life [5–8].

The persistence of conduct problems in these young children has implications for their developmental trajectory, including the emergence of comorbid mental disorders, social problems, and future maladaptation [9, 10].

Early intervention is crucial to improve these children’s outcomes. At the early stages of development, troubling signs (e.g., communication difficulties) can be targeted and treated with no need to wait for a full-blown diagnosis. This approach allows treatment to reach toddlers with a wide range of neurodevelopmental difficulties, preventing interventions to be limited to particular diagnostic categories.

Parent-mediated interventions can have a large and sustained effect at a relatively low cost [11], improving a broad range of developmental domains [12]. Also, caring for children with neurodevelopmental disorders can be very stressful for parents, which could in turn lead to the lower effectiveness of interventions [13] and parental mental health problems [14]. Thus, it is of utmost importance to support the parents of these children in their task of promoting their children’s development [15, 16]. With this aim, evidence-based parenting interventions have been developed for children with ASD symptoms and for preterm children [12, 17]. However, these interventions are insufficiently available within the Spanish Public Health System.

### Group interventions (the Incredible Years Autism Spectrum and Language Delays program: IY-ASLD®)

Group interventions show promise as a valuable resource to help parents cope with children’s behavioral, social, and emotional difficulties. This therapeutic approach has demonstrated effectiveness in terms of improving dysfunctional parenting styles, reducing children’s behavioral problems [18] and increasing parents’ ability to facilitate their children’s communication skills and vocabulary [19, 20]. Group interventions also provide social support for parents [21, 22]. This is especially important for parents who are more likely to experience depression and stress during the early years of their child’s life. This is the case for parents of children with ASD [23, 24] and those of preterm children [25], who have identified a need for parenting support to promote their children’s development [26].

The Incredible Years® parenting programs [27] are a set of interventions recommended by the NICE guidelines [28], primarily focused on strengthening the parent-child interaction and improving parenting skills in order to prevent or reduce children’s behavioral problems. The effectiveness of the Incredible Years program has been widely demonstrated in multiple randomized controlled trials, showing an improvement in terms of parental stress levels, depression, and parental coping, as well as in children’s behavior difficulties [29, 30]. A range of developmentally appropriate interventions for different age groups is offered.

The IY-ASLD® program has been specifically developed to target the needs and concerns of parents of children aged 2–5 years in the autistic spectrum or with language delays [15]. The program encourages positive parent-child relationships to promote children’s emotion regulation, social competence, language skills, school readiness, and peer relationships. The intervention teaches parents how to play in a child-directed way but with a specific focus on encouraging children’s communication and social engagement. It also focuses on how to use positive discipline to set limits and handle misbehavior. A pragmatic randomized controlled trial has been recently published, supporting the feasibility of delivering this intervention in the UK National Health Service (NHS) and showing good levels of acceptability, compliance, and fidelity to the program [31].

The IY-ASLD® program has never been trialed in Spain, where the Public Health System has shown a lack of resources to perform evidence-based early interventions for neurodevelopmental problems [32]. Thus, the validation and implementation of group interventions such as the IY-ASLD® program are a priority in terms of offering Spanish families better and earlier interventions that are brief, intensive, cost-effective, and based on scientific evidence.

### Aims and hypotheses

The main aim of the present study is to examine the feasibility of implementing the IY-ASLD® program in the Spanish Public Health System. Secondarily, we aim to explore initial evidence of the effects of treating parents of preschool children with ASD and preterm children with communication and/or socialization difficulties.

In relation to the primary objective, the research hypotheses are as follows:

I. It is possible to recruit and randomize families to an intervention group and a TAU condition.
II. Parents accept to participate in the program.
III. Parents’ compliance with the program is acceptable (attending at least 15 of the 22 sessions and a minimum of 50% of parents finishing the intervention).
IV. Parents report acceptable levels of satisfaction with the program.
V. The program can be reliably implemented in the Spanish Public Health System.

In relation to the secondary objective, the research hypotheses are as follows:

I. Parents who received the intervention present reduced levels of parental stress when compared to the control group.
II. Parents who received the intervention show reduced levels of depressive symptoms and expressed emotions when compared to the control group.
III. Parents who received the intervention present an increase in their positive parenting skills when compared to the control group.
IV. Parents who received the intervention report a decrease in the levels of children’s externalizing and internalizing symptoms when compared to the control group.

In order to achieve these goals, we designed a controlled randomized pilot study that will be conducted within the Spanish Public Health System, where participating families will be randomly allocated to the intervention group (receiving the IY-ASLD® program) or to the treatment as usual condition (TAU) on a 1:1 ratio.

## Methods

This is a multicentric study that will be carried out in Child and Adolescent Mental Health Services from three hospitals in Spain: Hospital Materno-Infantil in Málaga (HMIM), Hospital General Universitario Gregorio Marañón in Madrid (HGUGM), and Hospital Sant Joan de Déu in Barcelona (HSJD). Eligible participants will be parents of children diagnosed with ASD and parents of preterm children with communication and/or socialization difficulties.

### Sample size

The IY-ASLD® program will be implemented in Child and Adolescent Mental Health Services at each site. In two of them (HMIM and HGUGM), parents of children with ASD will be recruited (*n* = 48), and in one site (HSJD), parents of preterm children with communication and/or socialization difficulties will be included (*n* = 24). Participating families will be randomly allocated to the TAU condition or to the intervention group within each site.

For the sample size calculation, we used the outcome parental stress, measured through the questionnaire Parental Stress Index Short Form (PSI-SF) [33]. This is a 36-item scale, with a range of 180 points. There is data showing that a decrease of 16.5 points in the total scale stress score can be seen after attending an IY program [34]. Given that this study will be conducted with parents of children presenting neurodevelopmental difficulties, we anticipated that the decrease in the PSI-SF score would be lower. We estimated the necessary sample size considering a power of 80%, *α* = 0.05, a difference between pre- and post-test of 10 points, and a standard deviation of 20 [35], using a paired-samples *t* test. We estimated 34 participants needed per arm of the study. Thus, we aim to recruit approximately 70 participants. The drop-out rate has not been included when calculating the sample size, considering that a sample of 70 participants is similar or larger than previous pilot or feasibility studies [31, 36] and in line with recommendations by the National Institute of Health Research [37]. Moreover, previous studies involving interventions with parents of children with neurodevelopmental disorders have reported low attrition rates [18, 31, 36].

### Eligibility criteria

Different inclusion criteria were introduced for each site, given that the study was conducted in specialist ASD units in the HMIM and HGUGM sites, and in a specialized unit for preterm children at risk of developmental disorders in the HSJD site.

The following are the inclusion criteria:

(a) HMlM and HGUGM sites: parents/caregivers of children diagnosed with ASD (clinical diagnosis performed by psychiatrists or clinical psychologists in the service, based on DSM-5 diagnostic criteria), (b) HSJD site: parents/caregivers of preterm children (< 37 weeks of gestational age) with communication and/or socialization difficulties (defined as Vineland-III scores below 1SD in any of the communication or socialization subdomains)
Children aged 2–5 years
Parents/caregivers showing good understanding of the Spanish language
Parents/caregivers consenting to take part in the study and signing the informed consent

The following are the exclusion criteria:

1. Attending another structured parenting program (focused on improving parental strategies to help their children with their developmental or regulation difficulties) during the intervention phase of the study
2. Children in the care of their local authority

### Recruitment

**Fig. 1:**
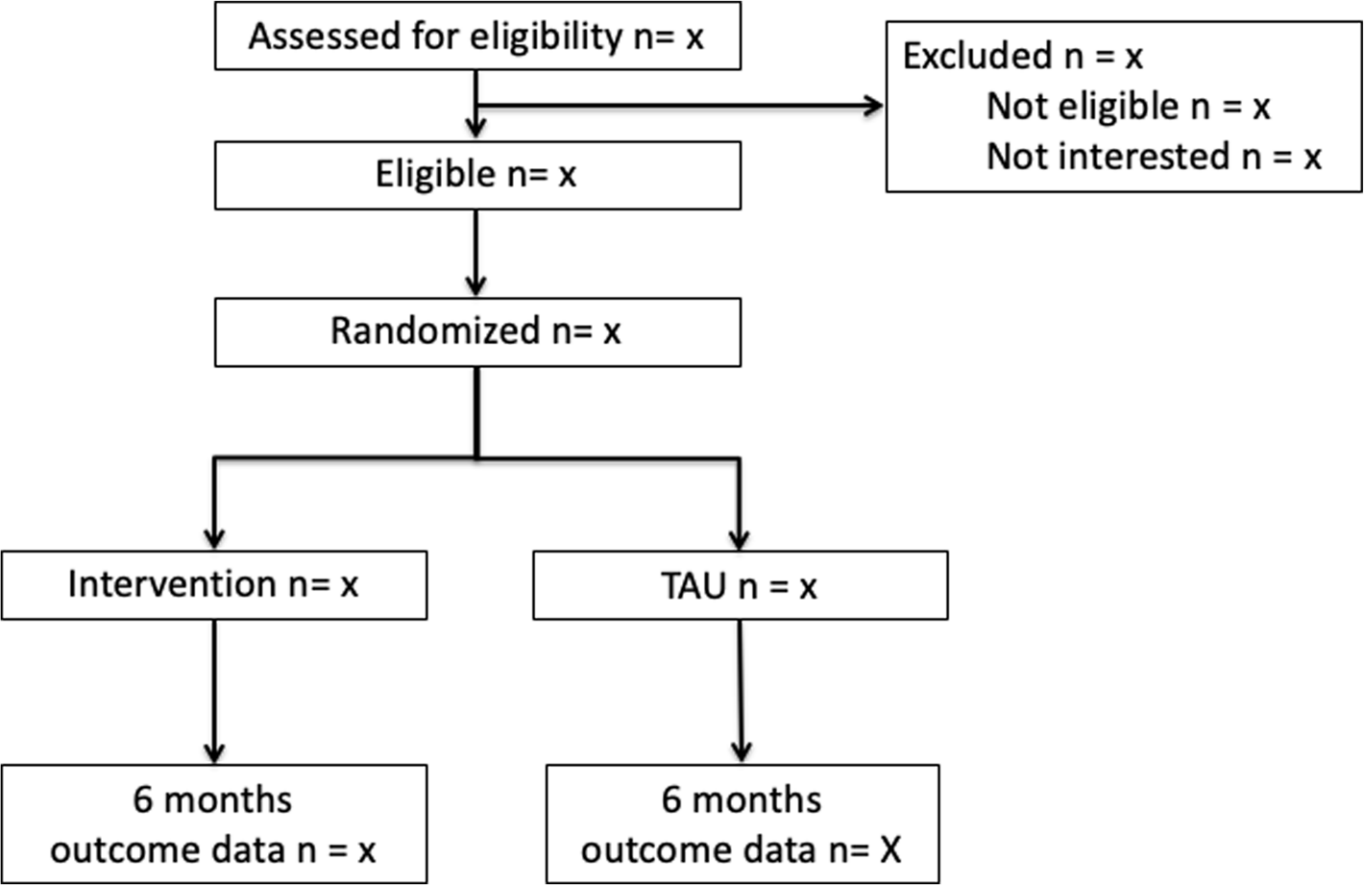
Participant flow diagram

Parents/caregivers will be recruited between January and March 2020 within the Child and Adolescent Mental Health Services of each hospital site, where patients have previously been assisted for their neurodevelopmental difficulties (Fig. 1). A leaflet with the inclusion and exclusion criteria will be handed out to clinicians working in the services. A short recruitment period has been anticipated, given that the study will be conducted in regional units with a high number of referrals and the study researchers work in close collaboration with clinicians. Clinicians will be asked to discuss the study with eligible families and will ask the families permission to be contacted by the study researchers. If they agree to be contacted about the project, a research assistant will call the family to discuss the study further. If the family is interested in participating in the study and meets the inclusion criteria, an appointment will be made with parents to sign the informed consent and to complete the pre-intervention assessment. During this visit, researchers will make sure that participants receive all the necessary information and have the opportunity to ask any questions. Participants will be able to discontinue the treatment sessions or drop out from the control group at any point at their request.

### Randomization

Randomization will take place once all the participating families have completed baseline measures. Then, they will be randomly allocated to the intervention group or to the TAU condition within each center. Randomization will be conducted using the Program R 3.6.2 (R Foundation for Statistical Computing), generating a replicable process fixing a random seed. Allocation will be stratified by developmental level, considering two levels above and below IQ = 70 (assessed with cognition subscale of the Developmental Profile-3) [38].

An independent researcher will carry out the randomization process, and researchers who are responsible for patient recruitment or for intervention delivery will not be involved. Due to the pragmatic and clinically focused nature of the study, further blinding procedures will not be possible.

### Intervention

The IY-ASLD® program is a 14-session group-based intervention for parents of children presenting neurodevelopmental difficulties. Due to the COVID-19 pandemic, the intervention will be adapted to be performed online in accordance with the Incredible Years developers, who will provide training and supervision to group leaders. The online format requires 22 sessions (approximately 6 months). Each online group will include approximately 5–6 participants. One of the main principles of the intervention is the collaborative approach, promoting parents’ discussion around the topics of interest and facilitating a solution-based perspective. The intervention includes video modeling and emphasizes the importance of practice-based learning through role-playing. The IY-ASLD® program takes into consideration the different developmental levels of each child and pairs parents according to this variable in role-play and other one-to-one discussions. Weekly home tasks will be assigned to parents, and families will be phoned each week to encourage home-based practice [15].

Fidelity to the treatment manual will be ensured in different ways. Group interventions will be conducted by experimented clinicians (child and adolescent psychiatrists and clinical psychologists), officially trained in the IY-ASLD® model by an accredited trainer. Group leaders will complete weekly checklists regarding fidelity to the intervention and participants will also complete weekly parent evaluations. Group leaders will be supervised by certified supervisors and will pursue accreditation according to the IY-ASLD® program standards.

The TAU condition involves outpatient appointments with a clinical psychologist or a psychiatrist, including unstructured interventions to foster children’s development and/or drug prescription when needed. Many families receive other interventions out of the National Health System (e.g., Early Development Centers), information that will be collected in the baseline assessment. Families allocated to the intervention group will also receive TAU.

### Measures (Table 1)

#### Baseline and sample descriptors

At baseline, the following sociodemographic and child clinical variables will be assessed: family structure, child’s age, gender, medical conditions, type and date of psychiatric diagnosis, psychotropic medication, educational status, current and past mental health interventions for parents and for the child, and neurodevelopmental problems in siblings. Socioeconomic status will be determined using Hollingdale’s Index of Social Position [39].

**Table 1:** Overview of the study timeline and measures

Time points				Study period		
	Enrolment	Allocation	Re-assessment	Weekly intervention (22 sessions)		Close-out
	January to February 2020	March 2020	September 2020	September 2020 Session 1	March 2021 Session 22	May 2021
**Enrollment:**						
Eligibility screen	**X**					
Informed consent	**X**					
Allocation		**X**				
**Intervention:**						
IY-ASLD® group	**X**	**X**	**X**	**X**	**X**	**X**
TAU group	**X**	**X**	**X**			**X**
**Assessments:**						
***Baseline variables:***						
Sociodemographic variables: Hollingdale’s Index of Social Position [39]	**X**					
The Modified Checklist for Autism in Toddlers-Revised (M-CHAT-R) [40]	**X**					
Social Communication Questionnaire (SCQ) [41]	**X**					
Vineland Adaptive Behavior Scale-III (parent/caregiver report form) [43]	**X**					
Developmental Profile-3 (DP-3) [38]	**X**					
***Outcome variables***:						
Parent Stress Inventory-Short Form (PSI-SF) [33]	**X**		**X**			**X**
Beck Depression Inventory (BDI) [46]	**X**		**X**			**X**
Alabama Parenting Questionnaire-Preschool version (APQ-Pr) [47]	**X**					**X**
Autism-Specific Five Minute Speech Sample (AS-FMFSS) [48]	**X**					**X**
Child Behavior Checklist (CBCL 1.5-5) [50]	**X**					**X**
ASD-P Parent Strategies Questionnaire [45]	**X**					**X**
Autism Program Parent Final Satisfaction Questionnaire [45]					**X**	**X**
Autism Program Parent Weekly Evaluation [45]				**X**	**X**	

*IY-ASLD®* Incredible Years Autism Spectrum and Language Delays program®, *TAU* treatment as usual

At baseline, the following questionnaires will be administered:

The Modified Checklist for Autism in Toddlers-Revised with Follow-up (M-CHAT-R/F) [40]: this tool is a parent-reported 20-item questionnaire screening ASD symptoms in children aged 24–30 months. This instrument will be administered before the intervention to describe children’s social communication difficulties. The Spanish translation has shown valid and reliable results.

Social Communication Questionnaire (SCQ) [41]: it is a 40-item parent report measure, yes/no format, based on the Autism Diagnostic Interview-Revised (ADI-R) [42]. The Lifetime version of this questionnaire will be administered before the intervention with the same aim as the M-CHAT-R, in children aged between 30 months and 5 years. It is a robust tool that has shown good validity, and it has been widely adopted by both the research and clinical community.

Vineland Adaptive Behavior Scale-III (VABS-III, parent/caregiver report form) [43]: it assesses adaptive functioning in different areas: communication (receptive, expressive, written), socialization (interpersonal/play and leisure time/coping), daily living skills (personal, domestic, community), and motor skills (fine and gross). It also generates a final adaptive composite score. This instrument will be used before the intervention to collect the level of functioning in different developmental areas. It has been considered a very efficient tool to measure the adaptive behavior profile of preschool children with developmental problems and shown an excellent test-retest reliability [44].

Developmental Profile-3 (DP-3) [38]: this is a 180-item parental questionnaire assessing developmental delays in different domains: motor, adaptative, socio-emotional, cognitive, and communication. It also computes an overall general development score. It will be used before the intervention to collect the child developmental level. It has shown good internal consistency.

#### Primary outcomes (feasibility)

Parents’ engagement with the program and participant retention will be monitored throughout, expecting they will attend at least 15/22 sessions with a minimum of 50% of parents finishing the program.

Autism Program Parent Weekly Evaluation [45]: this instrument is part of the IY-ASLD® program materials and will be used to collect information regarding compliance and satisfaction throughout the study.

Autism Program Parent Final Satisfaction Questionnaire [45]: this questionnaire is included within the IY-ASLD® program. It will be used to measure the acceptability and satisfaction with the intervention after the last session. It covers five areas: (1) the overall program, (2) usefulness of teaching format, (3) usefulness of specific teaching strategies, (4) evaluation of the group facilitators, and (5) the parent group.

Implementation fidelity will be ensured and monitored throughout the intervention process, following the strategies described above.

#### Secondary outcomes (preliminary effectiveness for parental outcomes)

ASD-P Parent Strategies Questionnaire [45]: this is a 60-item questionnaire included within the IY-ASLD® program. It is divided into 5 subdomains regarding the different strategies that will be learned throughout the program to promote social, emotional, language, and academic development, and how often parents use these strategies. This tool will be administered before and after the program implementation.

Parent Stress Inventory-Short Form (PSI-SF) [33]: this is a 36-item questionnaire that specifically focuses on assessing parental stress associated with the care of their offspring. It has three domains: parental distress, parent-child dysfunctional interaction, and difficult child, which combine to form a total stress scale. This tool will be administered before and after the intervention. It has shown good internal consistency.

Beck Depression Inventory (BDI) [46]: this is a 21-item screening tool assessing the severity of depressive symptoms. It is a standardized and validated questionnaire, often used in mood disorder assessments. It will be collected before and after the intervention. It has good reliability.

Alabama Parenting Questionnaire-Preschool revision (APQ-Pr) [47]: this is a 32-item parent-reported questionnaire measuring parenting practices that are consistently associated with disruptive child behaviors. This version has 3 dimensions: positive parenting, inconsistent parenting, and punitive parenting. It will be collected before and after the intervention. This measure has shown good internal consistency and validity.

Autism-Specific Five Minute Speech Sample (AS-FMFSS) [48]: this is a narrative 5-min interview used to measure parental-expressed emotions for children with ASD and related disorders*.* Parents are asked to speak about their child and the parent-child relationship —“I’d like you to speak for 5 minutes, telling me what kind of person (child name) is and how the two of you have got along together over the past 6 months.” Speech samples are audiotaped, transcribed, and coded following four global categories: (a) initial statement, (b) warmth, (c) relationship, (d) emotional over-involvement, (e) critical comments, and (f) positive comments. Expressed emotions will be measured before and after the intervention. Benson et al. [49] assessed 30 randomly selected speech samples by three different raters. Inter-rater reliability and code-recode reliability on two separate occasions, for all six AS-FMSS components and for total EE score, were both in the good to excellent range.

#### Other outcomes (preliminary effectiveness for child outcomes)

Child Behavior Checklist (CBCL 1.5-5) [50]: this is a parent-reported 99-item inventory that addresses specific externalizing and internalizing behaviors. The sum of scores forms a total problem score, and it also includes scores for externalizing and internalizing difficulties subdomains. It will be collected before and after the intervention. It has shown good internal consistency, also in children diagnosed with ASD [51].

### Data collection

Data will be collected at baseline (before performing randomization), right before the beginning of the intervention and after finishing the IY-ASLD® intervention. Due to the COVID-19 pandemic, the intervention has been postponed 6 months (from March 2020 to September 2020), and families will be re-assessed before the start of the group for an updated measure of parental depressive symptoms and stress. Parents consenting to participate (mother, father, or both) will be offered a hospital appointment before and after the intervention with a research assistant to complete the assessment, including self-report measures, children outcome measures, and a voice recording for the ASFMSS tool. If both parents participate in the study, they will fill out the children’s outcome measures together (by consensus) and the parental outcome measures individually. Those parents who discontinue or deviate from the intervention protocols will receive a telephone call from the research assistant to arrange further hospital appointments or to check if they are willing to complete the questionnaires for the main outcomes on the phone.

The timeline for participants’ enrollment, data collection, and the conduction of the intervention are scheduled in Table 1.

### Data analysis

Descriptive analysis will be used for primary outcomes (e.g., recruitment rates, number of sessions attended by participants, proportion attending at least 15 out of 22 sessions, and proportion finishing the program). Attrition and satisfaction rates with the program will be also described.

Differences (if any) in baseline and descriptive variables between intervention and TAU conditions will be described. Between-site differences will be analyzed with the chi-square and independent samples *t* tests and will be controlled for in the main analyses.

For secondary outcomes, the analysis will follow an intention-to-treat basis. Regarding parental stress and depression, the assessment taken right before the intervention begins will be the one included in the analysis. Differences between the treatment groups in terms of parental levels of stress, expressed emotions, and parenting practices will be determined with the analysis of covariance (ANCOVA). Relevant confounding variables, such as socio-demographic variables, cognitive level at baseline, or treatment site, will be added as covariates. We will also estimate 95% confidence intervals and effect sizes [52]. Further secondary analysis will use similar methods to compare treatment groups regarding additional child outcomes (i.e., CBCL externalizing problems).

Missing data (i.e., missing item responses in questionnaires) will be treated following the instructions stipulated in the questionnaire’s manual. Imputation methods will be considered for full-case missing data. The analysis will be conducted using the SPSS/STATA statistical packages.

### Data monitoring

The study does not involve any anticipated risks for participants, and therefore, there is not an external Data Monitoring Committee involved. There are no stopping guidelines for the trial, but participants can withdraw consent at any time.

### Trial status

The protocol for the current pilot study was registered in ClinicalTrials.gov (ID number: NCT04358484. Unique Protocol ID: PIC-220-19) and it was released in April 2020. The study began in January 2020, and the anticipated finish date is May 2021. The recruitment process was carried out during the same period in the 3 sites, from 3 February 2020 to 2 March 2020. Due to the COVID-19 pandemic, the intervention has been postponed to September 2020, and baseline measures (parental depressive symptoms and stress) will be repeated before the intervention begins.

## Ethics

The study has received ethical approval from the Ethics Committee at each site (PIC-220-19): CEIm Fundaciò Sant Joan de Déu (Barcelona), CEI provincial (Málaga), and CEIm Hospital Gregorio Marañón (Madrid). The trial data set will be stored in institutional encrypted computers and will itself be encrypted with a password. Only the staff involved in the study will be authorized to access the data. Participants will be informed about all protection data procedures and will be able to disengage from the study at any point, without affecting their usual clinical care. Written information regarding these aspects will be handed, and contact details from research assistants will be offered in order to clarify any doubts or worries participants might have.

Participants will be informed of the potential benefits and risks of participating in the study. Due to the nature of the study, it is unlikely that taking part in the research will result in any damage. After families are provided with sufficient information about the project, they will be asked to sign the written informed consent.

The study will be conducted following the principles developed by the World Medical Association, outlined in the Declaration of Helsinki.

## Dissemination plans

The results of this study will inform the acceptability and feasibility of implementing, and culturally adapting, the IY-ASLD® intervention for Spanish families. The results will be described in a final report to the funder, and they will be published in peer-reviewed journals.

## Discussion

The main aim of the study is to pilot the IY-ASLD® intervention in Public Mental Health Services in Spain. The current study has several strengths. Firstly, to the best of our knowledge, this is the first time that this program will be conducted in Spain. This is relevant in terms of improving the availability of treatments for children with neurodevelopmental difficulties within the Spanish Public Health System. Also, given that this intervention has been designed and tested in other countries (the USA and the UK), it is important to test if it can be implemented in Spain, where we have language, cultural, and healthcare system differences. Moreover, the IY-ASLD® program will be tested in different areas within Spain, providing a broad view of its feasibility across the country. Secondly, the present study targets a broad range of patients with early neurodevelopmental problems, as not only those meeting the diagnostic criteria for ASD are included, but parents of premature children with socialization and/or communication difficulties are also offered the intervention. Different strategies have been put into place in order to provide coherence and internal consistency to the study. Group leaders have attended the official training and will be following the procedures established in the program to assure fidelity to the intervention. Finally, preliminary efficacy will be analyzed, which could inform the importance of conducting a larger randomized controlled trial. Regarding limitations, the sample size might be limited in terms of power for some effect analyses, considering that the trial has not been powered for between-group differences. However, it is of note that we are introducing validated measures that go beyond parent-reported questionnaires to assess parent-child relationships (i.e., ASFMFSS), which is of paramount importance when evaluating parenting interventions and children in preschool years. Another limitation is the repetition of the assessment of parental stress and depression (due to the COVID-19 pandemic) once parents already know their group allocation.

This trial will represent the first step to plan future projects. Positive results regarding feasibility (primary outcome) will be required to conduct a larger RCT. The limited sample size might not allow detecting significant differences regarding preliminary efficacy (secondary outcome). A larger RCT could guide policymakers to fund and further implement the intervention within the Spanish Public Health System.

## Supplementary Information

**Additional file 1.** Consent form for participants.

**Additional file 2.** Information sheet for participants.

## Data Availability

As mentioned above, the trial data set will be stored in institutional encrypted computers and will itself be encrypted with a password. Only the staff involved in the study will be authorized to access the data. The data sets analyzed during the current study are available from the corresponding author on reasonable request.
