# Supplementary material for: Protocol for a randomized pilot study (FIRST STEPS): implementation of the Incredible Years-ASLD® program in Spanish children with autism and preterm children with communication and/or socialization difficulties
Source: Trials. 2021 Apr 20;22:291. doi: 10.1186/s13063-021-05229-1 (PMC8056105; doi:10.1186/s13063-021-05229-1)
Supplement: Supplementary file 1 — Additional file 1. Consent form for participants. [file 13063_2021_5229_MOESM1_ESM.docx]

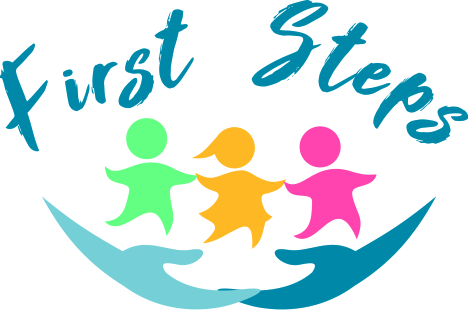

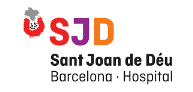


**Unidad de Investigación**

**Consentimiento informado para estudio científico de investigación**


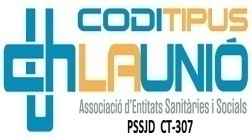


Título del Proyecto First Steps: estudio piloto aleatorizado para la implementación del programa de parentalidad Incredible Years® en preescolares con autismo y retraso del lenguaje en España.

Nombre Apellidos

Nº. HC Edad *_____ años* DNI

Passeig de Sant Joan de Déu, 2 08950. Esplugues de Llobregat, Barcelona

**DECLARO:** *que la doctora – Laia Villalta* colegiado/a número  *44744* me ha propuesto participar en el estudio de investigación First Steps: estudio piloto aleatorizado para la implementación del programa de parentalidad Incredible Years**®** en preescolares con autismo y retraso del lenguaje en España. Tras recibir la información correspondiente, manifiesto que:

**1.** He recibido la hoja informativa y he comprendido la información sobre el estudio en el que participaré.

**2**. He sido informado/a de las implicaciones derivadas de la participación.

**3**. Soy consciente de que mi participación es voluntaria y puedo retirarme en el momento que decida sin tener que dar explicaciones y sin que repercuta en mi atención.

**4**. He sido informado/a del tratamiento de datos del Hospital Sant Joan de Déu, de sus plazos de conservación y de su utilización para investigación por parte del investigador/a principal del proyecto y que en cualquier momento puedo ejercer mis derechos de Acceso, Rectificación, Supresión/Cancelación y Oposición en los términos y condiciones establecidos por la normativa vigente en materia de Protección de Datos (LOPD vigente, RGPD-UE 679/2016), como por ejemplo solicitar mis datos personales, rectificarlos si fuera necesario, así como revocar en cualquier momento la autorización de inclusión en el estudio. Para ejercer estos derechos hay que dirigirse, personalmente o por escrito, al Investigador/a principal o a la Unidad de Atención al Usuario del centro, indicando claramente la petición, refiréndose a este estudio y adjuntando copia de documento identificativo (DNI/NIE). Dirección del Centro: Hospital Sant Joan de Déu (HSJD), Paseo de Sant Joan de Déu, 2 08950 Esplugues de Llobregat (Barcelona)-España. Responsable de Tratamiento: HSJD. En caso de disconformidad con el tratamiento de los datos o con el ejercicio de los derechos correspondientes puedo dirigirme por escrito al Delegado de Protección de Datos del Hospital (dpd@sjdhospitalbarcelona.org) a la dirección previamente indicada o reclamar directamente ante las Autoridades de Control (Autoridad Catalana de Protección de Datos o la Agencia Española de Protección de Datos). Este documento y los datos recogidos en el estudio se conservarán bajo la custodia del HSJD por un periodo no inferior a 10 años.

He entendido las explicaciones que me han facilitado en un lenguage claro y sencillo y el/la investigador/a que me ha atendido me ha permitido realizar todas las observaciones y me ha aclarado todas las dudas que he planteado.

Y en tales condiciones,

**SI NO**

**DOY MI CONSENTIMIENTO** para participar en el estudio de investigación First Steps: estudio piloto aleatorizado para la implementación del programa de parentalidad Incredible Years® en preescolares con autismo y retraso del lenguaje en España.  *Barcelona*, a

**Firma del/de la representante/tutor/a Firma del/de la investigador/a**

**DNI y nº colegiado/a**

***Familiar o allegado/a del/de la paciente, representante legal.**

***El orden de la relación para la autorización es el siguiente: paciente, cónyuge, padres, hijos/as, hermanos/as, familiares o personas allegadas y tutores/oras.**

Nombre Apellidos

Edad *_____ años*  DNI

En calidad de*
