# Supplementary material for: Protocol for a randomized pilot study (FIRST STEPS): implementation of the Incredible Years-ASLD® program in Spanish children with autism and preterm children with communication and/or socialization difficulties
Source: Trials. 2021 Apr 20;22:291. doi: 10.1186/s13063-021-05229-1 (PMC8056105; doi:10.1186/s13063-021-05229-1)
Supplement: Supplementary file 2 — Additional file 2. Information sheet for participants. [file 13063_2021_5229_MOESM2_ESM.docx]

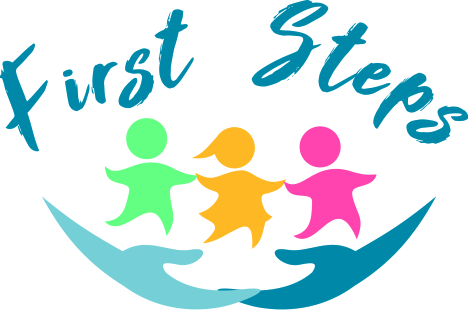


**HOJA INFORMATIVA**

**First Steps: estudio piloto aleatorizado para la implementación del programa de parentalidad Incredible Years® en preescolares con autismo y retraso del lenguaje en España.**

Apreciado/a Sr./Sra.,

Le proponemos participar en el estudio: First Steps: estudio piloto aleatorizado para la implementación del programa de parentalidad Incredible Years**®** en preescolares con autismo y retraso del lenguaje en España.

Antes de confirmar su participación en el estudio, es importante que entienda en qué consiste. Por favor, lea con atención este documento y haga todas las preguntas que considere oportunas.

OBJETIVOS DEL ESTUDIO

Los niños prematuros que presentan un retraso en el desarrollo del lenguaje, pueden tener mayores dificultades en regular sus emociones y su conducta. Todo ello puede hacer que su cuidado resulte particularmente estresante para los padres, y también que las intervenciones sean menos efectivas.

El programa *Incredible Years***®**, está destinado a padres de niños entre 2 y 5 años con retraso en el desarrollo del lenguaje, y tiene como objetivo mejorar los niveles de estrés y las habilidades de afrontamiento parental, así como el comportamiento negativo de los niños. En esta intervención grupal, además de aprender diversas estrategias para favorecer el desarrollo de habilidades en los niños, los padres reciben apoyo de otros padres y de los terapeutas. **Está enfocada a abordar precozmente las dificultades comunicativas y conductuales de los niños, potenciando los aspectos positivos de la relación padres-hijos, promoviendo el desarrollo del lenguaje, de la empatía, de las habilidades de socialización y de regulación emocional, así como la implementación de límites para el manejo de conducta.**

Existe amplia evidencia científica sobre la efectividad del programa *Incredible Years***®**, el cual está reconocido como la primera opción de tratamiento en países de habla inglesa (ej. Reino Unido), para niños con problemas de neurodesarrollo y dificultades conductuales. Con este estudio se pretende implementar este programa por primera vez en España.

PARTICIPACIÓN VOLUNTARIA

Su participación es totalmente voluntaria. La negativa a participar en la investigación no tendrá ninguna repercusión en su tratamiento. En cualquier caso, participe o no en el estudio, seguirá recibiendo el tratamiento habitual.

BENEFICIOS Y RIESGOS

Se espera que la intervención mediante la RCc puede beneficiar a los pacientes con DC. Se le informará de los resultados de dicha intervención.

Debe saber que del estudio se obtendrá información de gran interés científico para mejorar los tratamientos para pacientes con un primer episodio psicótico. Ni usted ni su hijo corre ningún riesgo esperado derivado de la participación en el estudio.

CONFIDENCIALIDAD Y PROTECCIÓN DE DATOS PERSONALES

Se garantiza la confidencialidad de los datos personales. Los resultados del estudio se almacenarán en archivos específicos creados específicamente para este fin y estarán protegidos con las medidas de seguridad exigidas en la legislación vigente. Ningún dato médico personal que permita su identificación será accesible a ninguna persona que no sea su médico/a, ni podrán ser divulgados por ningún medio, conservando en todo momento la confidencialidad médico/a-paciente. Los resultados obtenidos podrán ser consultados por los y las investigadoras del estudio y ser presentados en congresos nacionales e internacionales, así como publicados en revistas científicas, sin que consten los datos personales de los y las participantes. Si usted desea y una vez finalizado el estudio, le informaremos sobre los resultados obtenidos y el significado científico.

 En cualquier momento podrá ejercer sus derechos de Acceso, Rectificación, Cancelación/Supresión, Oposición (derechos ARCO) y cualquier otro derecho reconocido en los términos y condiciones establecidos por la legislación vigente en materia de Protección de Datos (LOPD vigente, Reglamento General de Protección de Datos de la Unión Europea, RGPD-UE, 679/2016), como por ejemplo solicitar sus datos personales, rectificarlos si fuera necesario, así como revocar la autorización de inclusión en el estudio. Para ejercer estos derechos debe dirigirse, personalmente o por escrito, al Investigador/a principal del estudio o a la Unidad de Atención al Usuario del Centro, indicando claramente su petición y adjuntando copia del documento identificativo (DNI/NIE). Dirección del Centro: Parc Sanitari San Joan de Déu (PSSJD), Dr. Antoni Pujadas 42, 08830 Sant Boi de Llobregat. El Responsable de Tratamiento (Actividades de Tratamiento del área de "BÚSQUEDA") es PSSJD. En caso de disconformidad con el tratamiento de sus datos o con el ejercicio de sus derechos puede dirigirse por escrito al Delegado de Protección de Datos de PSSJD (oficina_dpd@pssjd.org) a la dirección antes indicada, o reclamar directamente ante las Autoridades de Control (Autoridad Catalana de Protección de Datos: http://apdcat.gencat.cat/ca/contacte/apdcat@gencat.cat o Agencia de Protección de Datos, http://www.agpd.es/portalwebAGPD /CanalDelCiudadano/index-ides-idphp.php). Este documento y los datos personales recogidos y generados durante el estudio se conservarán bajo la custodia de PSSJD por un periodo no inferior a 10 años. Este estudio no genera decisiones automatizadas ni generación de perfiles, ni conlleva transferencia internacional de los datos personales recogidos y generados fuera del ámbito de protección legal de la Unión Europea.

PREGUNTAS / INFORMACIÓN

Si desea hacer una pregunta o aclarar algún tema relacionado con el estudio, o si precisa ayuda por cualquier problema de salud relacionado con el estudio, por favor, no dude en ponerse en contacto con el/la investigador/a principal del estudio:

Dr. / Dra. Ester Camprodon Rosanas Teléfono: _ +34 932804000. ext 80440__

El/La investigador/a principal le agradece su inestimable colaboración.

Estudio de nvestigación Terapia de estimulación cognitiva computarizada en adolescentes con un primer episodio psicótico: Ensayo Controlado Aleatorizado

PROCEDIMENTO Y DURACIÓN

Este es un estudio piloto para confirmar la viabilidad de implementar este programa en nuestro medio. Este estudio consta de dos grupos que se crearan al azar (Grupo A = tratamiento habitual + programa de parentalidad Incredible Years**®**; Grupo B = tratamiento habitual). Solamente los padres que sean asignados al Grupo A van a acudir al Hospital para realizar la intervención grupal durante 4 meses. Todos los padres (Grupo A y Grupo B) van a continuar con sus tratamientos habituales, y van a acudir a consultas externas de salud mental de nuestro hospital para la realización de 2 visitas a lo largo de 4 meses. En ambas visitas se va a realizar una evaluación estandarizada de la evolución del desarrollo de su hijo en el área lenguaje, junto a unos cuestionarios a rellenar por los padres. Se les entregará un informe con los resultados de la evaluación de su hijo para la coordinación con sus terapeutas y educadores habituales. El grupo A, además, participará en una terapia grupal durante 14 semanas, con sesiones semanales de aproximadamente 2 horas de duración.

Los terapeutas del grupo van a grabar un audio y dos de las sesiones terapéuticas en vídeo para optimizar el rigor y la fiabilidad de la terapia a través de la revisión de los contenidos implementados por parte de supervisores terapéuticos. La información digital será almacenada en ordenadores pertenecientes al sistema nacional de salud protegidos por claves personales a las que solamente los investigadores tendrán acceso. No se incluirá ningún tipo de información clínica o datos personales, más allá de lo que pueda observarse en la sesión terapéutica. La información digital se enviará por correo certificado a los supervisores y estará cifrada, de manera que solo ellos tendrán permiso para abrirlo al disponer de la clave correspondiente. Una vez el estudio esté finalizado se eliminará este material.

El equipo de investigación hará todo lo posible para responder cualquier pregunta o preocupación que le pueda surgir a lo largo del proceso. Durante las evaluaciones les entregaremos un número de contacto e email para poder contactar con nosotros fácilmente.

BENEFICIOS Y RIESGOS DE SU PARTICIPACIÓN EN EL ESTUDIO

Se espera que tras la participación en la intervención Incredible Years**®**, se reduzcan los niveles de estrés de los padres, que estos aprendan nuevas estrategias para fomentar aprendizajes en sus hijos, y que tengan más habilidades para ayudarles a regular sus emociones y sus conductas.

Por las características del estudio, no existen riesgos asociados a la participación en el mismo. Solamente habrá un compromiso de su tiempo como familia, ya que la valoración del desarrollo de su hijo y los cuestionarios parentales les implicará asistir a nuestro hospital durante una mañana o tarde.

¿QUE PASARÁ CON SU INFORMACIÓN?

El Hospital Sant Joan de Déu (Calle Sant Joan de Déu nº 2 08950 Esplugues de Llobregat, Barcelona) como responsable del tratamiento de sus datos, le informa que el tratamiento, la comunicación y la cesión de los datos de carácter personal de todos los participantes se ajustará a la legislación vigente (Reglamento Europeo UE 2016/679 y Ley Orgánica 3/2018 de 5 de diciembre de Protección de Datos Personales).

Los datos para este estudio se recogerán identificados únicamente mediante un código, por lo que no se incluirá ningún tipo de información que permita identificar a los participantes. Sólo el médico del estudio y sus colaboradores podrán relacionar los datos recogidos en el estudio con su historia clínica. Su identidad no estará al alcance de ninguna otra persona a excepción de una urgencia médica o requerimiento legal. Podrán tener acceso a su información personal identificada, las autoridades sanitarias, el Comité de Ética de Investigación y personal autorizado por el promotor del estudio, cuando sea necesario para comprobar datos y procedimientos del estudio, pero siempre manteniendo la confidencialidad de acuerdo a la legislación vigente.

Sólo se cederán a terceros y a otros países los datos codificados, que en ningún caso contendrán información que pueda identificar al participante directamente (como nombre y apellidos, iniciales, dirección, número de la seguridad social, etc.). En el supuesto de que se produjera esta cesión, sería para la misma finalidad del estudio descrito y garantizando la confidencialidad. No se tomarán decisiones automatizadas de sus datos ni se elaborarán perfiles. Si se realizara una transferencia de datos codificados fuera de la UE, ya sea a entidades relacionadas con el centro hospitalario donde usted participa, a prestadores de servicios o a investigadores que colaboren con su médico, sus datos quedarán protegidos por salvaguardas como contratos u otros mecanismos establecidos por las autoridades de protección de datos.

Usted puede ejercer los derechos de acceso, modificación, oposición, supresión, limitación del tratamiento y portabilidad (solicitar una copia o que se trasladen a un tercero) de los datos que ha facilitado para el estudio. Para ejercitar estos derechos, o si desea saber más sobre confidencialidad, deberá dirigirse al investigador principal del estudio o al Delegado de Protección de Datos del Hospital Sant Joan de Déu a través de dpd@sjdhospitalbarcelona.org. Asimismo tienen derecho a dirigirse, si no quedara satisfecho/a, a la Autoritat Catalana de Protecció de dades (http://apdcat.gencat.cat/ca/contacte/apdcat@gencat.cat) o a la Agencia de Protección de Datos (http://www.agpd.es/portalwebAGPD/CanalDelCiudadano/index-ides-idphp.php)

Los datos ya recogidos no se pueden eliminar aunque usted abandone el estudio, para garantizar la validez de la investigación, pero no se recogerán nuevos datos si usted decide dejar de participar.

El Investigador y el Promotor conservarán los datos recogidos para el estudio al menos hasta unos años tras su finalización. Posteriormente, la información personal solo se conservará por el centro para el cuidado de su salud y por el investigador o promotor para otros fines de investigación científica si el paciente hubiera otorgado su consentimiento para ello, y si así lo permite la ley y requisitos éticos aplicables.

Si da su consentimiento, los datos recogidos durante este estudio, podrían utilizarse para futuros proyectos de investigación relacionados con éste, siempre manteniendo la confidencialidad tal como se ha expuesto.

Los resultados de la investigación podrán formar parte de publicaciones científicas guardando la confidencialidad.

Si está interesado en participar, el referente del programa le entregará la hoja de consentimiento informado.

**Versión 2.2. 18/12/2019**
